# Efficient electrical control of thin-film black phosphorus bandgap

**DOI:** 10.1038/ncomms14474

**Published:** 2017-04-19

**Authors:** Bingchen Deng, Vy Tran, Yujun Xie, Hao Jiang, Cheng Li, Qiushi Guo, Xiaomu Wang, He Tian, Steven J. Koester, Han Wang, Judy J. Cha, Qiangfei Xia, Li Yang, Fengnian Xia

**Affiliations:** 1 Department of Electrical Engineering, Yale University, 15 Prospect St Becton 519, New Haven, Connecticut 06511, USA; 2 Department of Physics, Washington University, St Louis, Missouri 63130, USA; 3 Department of Mechanical Engineering and Materials Science, Yale University, New Haven, Connecticut 06511, USA; 4 Department of Electrical and Computer Engineering, University of Massachusetts, Amherst, Massachusetts 01003, USA; 5 Ming Hsieh Department of Electrical Engineering, University of Southern California, Los Angeles, California 90089, USA; 6 Department of Electrical and Computer Engineering, University of Minnesota, Minneapolis, Minnesota 55455, USA

## Abstract

Recently rediscovered black phosphorus is a layered semiconductor with promising electronic and photonic properties. Dynamic control of its bandgap can allow for the exploration of new physical phenomena. However, theoretical investigations and photoemission spectroscopy experiments indicate that in its few-layer form, an exceedingly large electric field in the order of several volts per nanometre is required to effectively tune its bandgap, making the direct electrical control unfeasible. Here we reveal the unique thickness-dependent bandgap tuning properties in intrinsic black phosphorus, arising from the strong interlayer electronic-state coupling. Furthermore, leveraging a 10 nm-thick black phosphorus, we continuously tune its bandgap from ∼300 to below 50 meV, using a moderate displacement field up to 1.1 V nm^−1^. Such dynamic tuning of bandgap may not only extend the operational wavelength range of tunable black phosphorus photonic devices, but also pave the way for the investigation of electrically tunable topological insulators and semimetals.

## Abstract (web summary)

Layered black phosphorous has gained significant attention in the 2D materials community, and dynamical control of its bandgap is key to enable novel applications. Here, the authors demonstrate continuous electrical bandgap tuning using moderate displacement fields.

Bandgap is a fundamental material parameter that governs the transport and light–matter interaction properties. Black phosphorus (BP) lately emerged as a promising layered material^1^,^2^,^3^,^4^,^5^,^6^,^7^,^8^,^9^,^10^, which bridges the gap between the gapless graphene^11^,^12^ and semiconducting transition metal dichalcogenides (TMDCs)^13^ such as molybdenum disulfide (MoS_2_) with a relatively large bandgap of around 2 eV. Owing to the strong electronic state coupling among layers in BP, its direct bandgap varies significantly from around 0.3 eV in bulk to 2 eV in monolayer form^9^. On the other hand, an approach that can dynamically tune the bandgap of BP to well below 0.3 eV is of great scientific and technological importance. In fact, the feasibility of bandgap tuning in few-layer BP using an electric field has been explored theoretically^14^,^15^,^16^,^17^. Recently, photoemission spectroscopy experiments in doped few-layer BP through the adsorption of potassium atoms show that indeed its bandgap can be tuned and even completely closed^18^. However, the estimated electric field induced by potassium adsorption in few-layer BP to widely tune its bandgap is on the order of several volts per nanometre. Such a large electric field prevents the realization of bandgap tunable electronic and photonic devices. Moreover, heavily doped material is usually not ideal for device applications. As a result, it is highly attractive to realize a widely tunable bandgap in intrinsic BP with a moderate field accessible with regular dielectrics.

In this work, we demonstrate bandgap tuning in BP using an electric field directly and discover unique thickness-dependent bandgap tuning properties, guided by first-principles calculations. In a 4 nm-thick intrinsic BP film, the bandgap tuning is limited to around 75 meV and shows strong nonlinear dependence on the biasing field. This peculiar field dependence is due to the strong interlayer electronic-state coupling and is captured by density functional theory (DFT) calculations very well. On the contrary, in a 10 nm-thick intrinsic BP in which the film is thick enough such that the field-induced potential difference can overcome the interlayer coupling, we demonstrate the efficient, continuous tuning of its bandgap from ∼300 to below 50 meV with an external displacement field up to 1.1 V nm^−1^. We further show that in this case, the additional dielectric screening effect beyond a simple DFT prediction due to the significant bandgap shrinkage has to be taken into account to describe the experimental results.

## Results

### Device fabrication and basic characterizations

**Figure 1:**
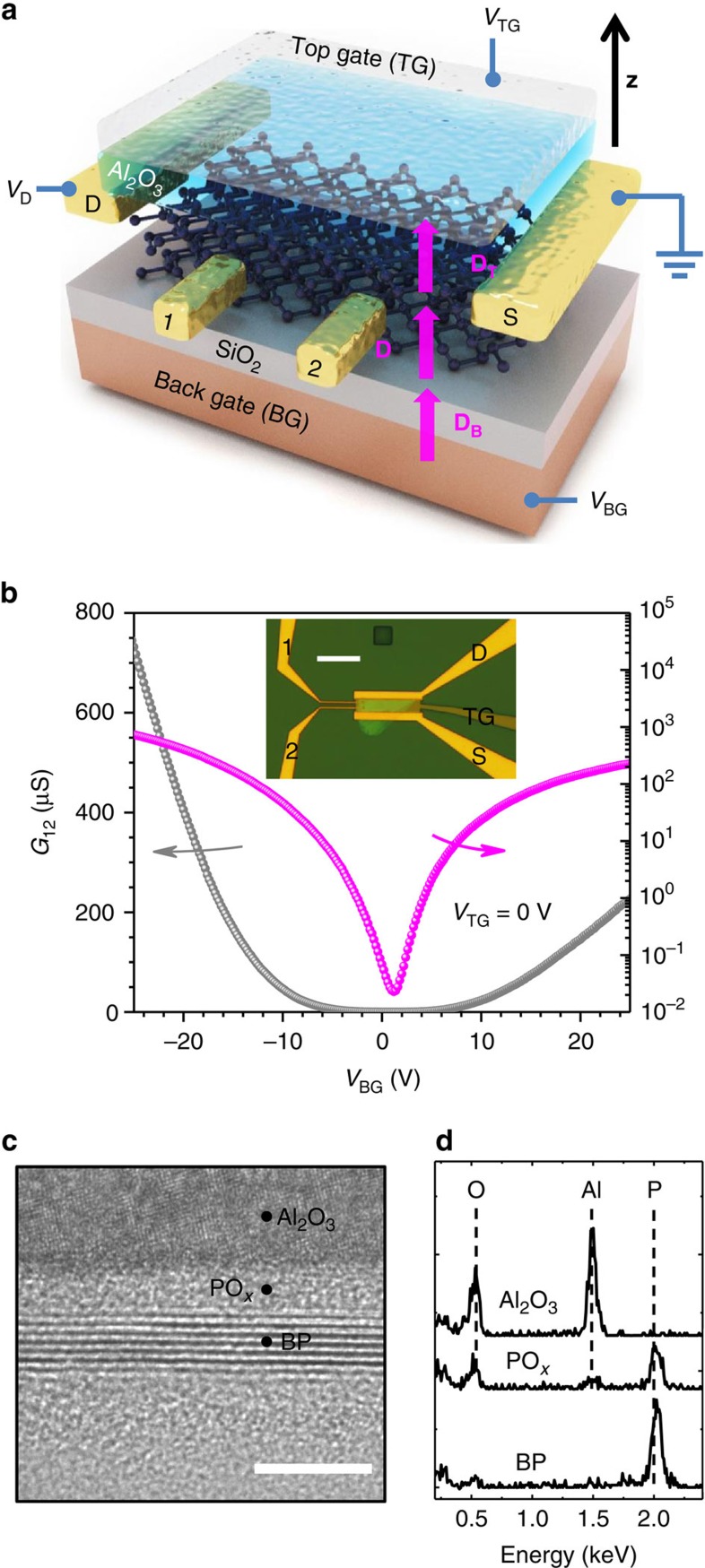
Experimental scheme for BP bandgap tuning. (**a**) The schematic view of the dual-gate BP thin-film transistor for the realization of bandgap tuning. (**b**) The 4 nm-thick BP film conductance in linear (left axis) and logarithmic (right axis) scales as a function of the back gate bias (*V*_BG_) at zero top gate bias (*V*_TG_=0 V). The conductance *G*_12_ is measured using a four-probe scheme as shown in **a**, to eliminate the effects of the contact resistance. Inset: an optical image of a typical dual-gate BP transistor. Scale bar, 10 μm. (**c**) High-resolution cross-section transmission electron micrograph showing the BP layer, which is about 4 nm thick. The PO_*x*_ layer between the BP layer and Al_2_O_3_ is 2–3 nm thick. Scale bar, 8 nm. (**d**) Energy dispersive X-ray spectra from three selected spots in **c**.

We use dual-gate BP transistors as shown in Fig. 1a for the bandgap measurement under a bias field. Here, the thin-film BP channel is sandwiched between the back (90 nm-thick silicon oxide) and top (24 nm-thick aluminum oxide) gate dielectrics. The source and drain leads are made from chromium/gold (3/30 nm). The top gate is made from titanium/platinum (1/10 nm) and the silicon substrate is used as the back gate. The bandgap is determined through the BP conductance at the charge neutrality point under a vertical biasing field. To eliminate the impact of metal/BP contact on the conductance measurement, a four-probe scheme is adopted, as shown in Fig. 1a. In our measurements, the source is grounded and the drain bias *V*_D_ is kept at 100 mV. From the channel current and the voltage difference between probes 1 and 2, we determine the conductance of the BP film. The detailed device fabrication and measurement procedures are discussed in the Methods section. In Fig. 1b, the measured conductance *G*_12_ of a 4 nm-thick BP flake between probes 1 and 2 (see inset) at room temperature is plotted as a function of the back gate bias, *V*_BG_, whereas the top gate bias, *V*_TG_, is set to be 0 volt. The conductance is plotted in both linear (left axis) and logarithmic (right axis) scales. As the bandgap of BP is moderate, the conductance in the insulating state at room temperature can still be measured using a four-probe scheme, as shown by the logarithmic plot (right axis, Fig. 1b). Here the BP thickness is determined using the atomic force microscopy (AFM) together with the cross-section high-resolution transmission electron microscopy, as shown in Supplementary Fig. 1 and Fig. 1c, respectively. Energy dispersive X-ray spectra from three selected spots in Fig. 1c are plotted in Fig. 1d. It is rather clear that the phosphorus oxide (PO_*x*_) at the top BP surface is 2–3 nm thick due to the exposure to external environment during processing. However, at the bottom BP/SiO_2_ interface as shown in Fig. 1c, there is no obvious sign of BP oxidization, because the sample exfoliation was performed in the glove box. As a result, the intrinsic BP thickness in our devices is always 2–3 nm less than the value measured using AFM, as discussed in Supplementary Note 1. In fact, our previous generations of BP devices have thicker PO_*x*_ on both top and bottom surfaces due to the exfoliation in ambient environment and lack of top gate dielectric protection.

### Bandgap tuning in a 4 nm-thick BP film

**Figure 2:**
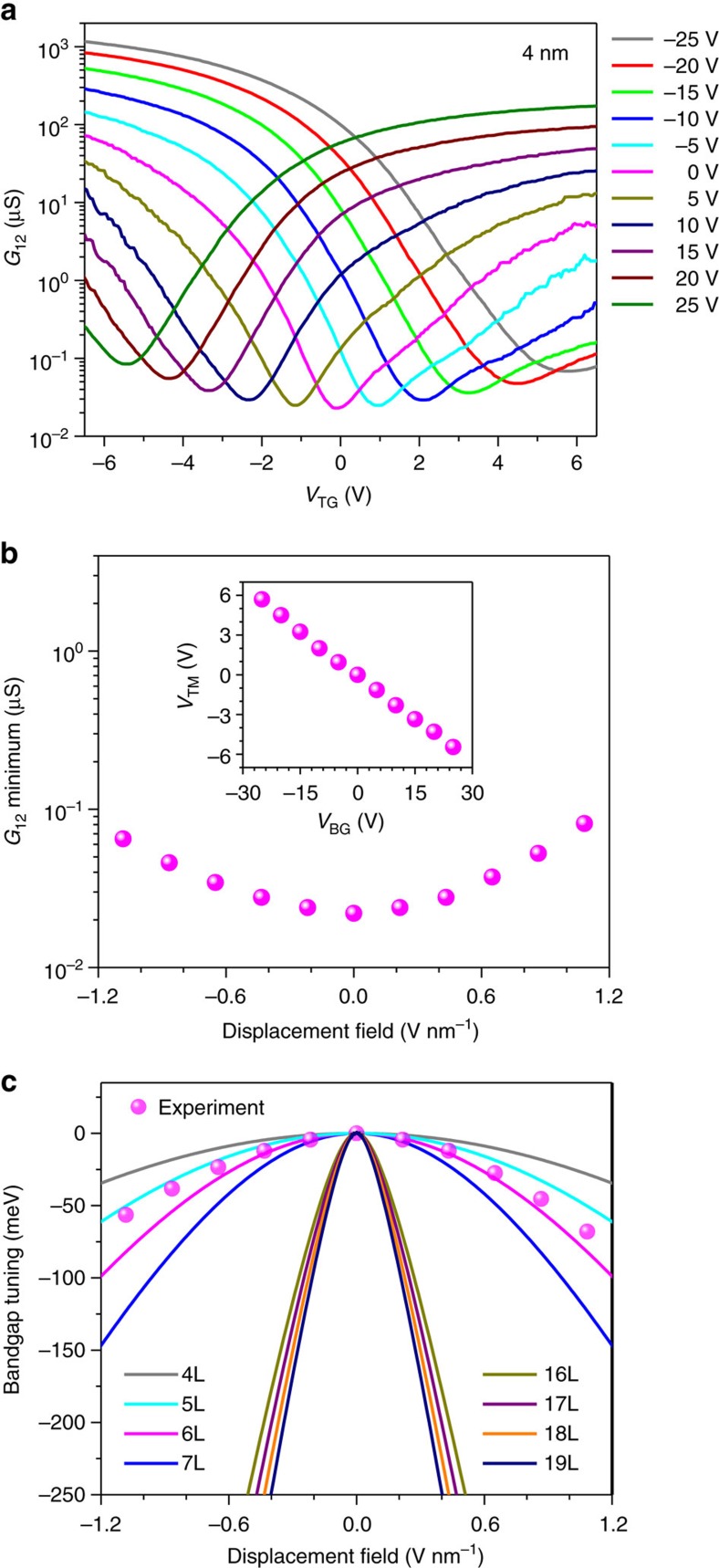
Bandgap tuning in a 4 nm-thick BP film. (**a**) The 4 nm-thick BP film conductance as a function of top gate bias (*V*_TG_) at different static back gate biases (*V*_BG_) from −25 to 25 V. (**b**) The minimum conductance at the charge-neutrality point as a function of external displacement field. Inset: the top gate bias at which the minimum conductance occurs (*V*_TM_) as a function of the back gate bias (*V*_BG_). The linear relation indicates the effective doping compensation by the back and top biases, leading to an insulating state at the charge-neutrality. (**c**) Solid lines: The calculated bandgap tuning properties for BP films consisting of 4, 5, 6, 7, 16, 17, 18 and 19 layers using a tight-binding model built on the DFT. The BP films with different thicknesses exhibit distinctively different tuning properties. Purple dots: the measured bandgap tuning results for the 4 nm-thick BP film (∼7 layers).

We further performed conductance measurements in such dual-gated BP transistors by sweeping the top gate bias at different static back gate biases. The measurement results for a BP transistor with a channel thickness of around 4 nm are summarized in Fig. 2a. As shown in the inset of Fig. 2b, the top gate bias (*V*_TG_) at which the conductance is minimized (*V*_TM_) scales almost linearly with the back gate bias (*V*_BG_). This observation indicates that the top gate bias with opposite sign can effectively compensate the doping introduced by the back gate at the *V*_TG_=*V*_TM_, leading to an insulating state at the charge neutrality point. As the source is grounded and a very small drain bias of 100 mV is applied, the displacement fields in the back (**D**_B_) and top (**D**_T_) gate dielectric are 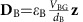 and 
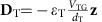
, respectively, where *ɛ*_B_(∼3.9) is the relative permittivity of SiO_2_, *d*_B_ (90 nm) is the thickness of SiO_2_, *ɛ*_T_ is the overall relative permittivity of top gate dielectric, *d*_T_ (26 nm) is the total thickness of top gate dielectric and **z** is the unit vector along the vertical direction as shown in Fig. 1a. Here, displacement is defined as positive when it points from back towards the top gates. The *d*_T_ includes the 2 nm-thick PO_*x*_ layer and 24 nm-thick Al_2_O_3_. From the slope of the curve in the inset of Fig. 2b, *ɛ*_T_ is determined to be ∼5.6. At the charge-neutrality condition (*V*_TG_=*V*_TM_), **D**_B_=**D**_T_=**D**, where **D** is the displacement field in BP channel, as shown in Fig. 1a. We assume that at the charge-neutrality condition, the free carrier concentration in BP is minimized such that **D** is approximately uniform across the entire channel. For 100 mV source-drain bias, the maximum possible displacement variation between the BP and the top gate along the channel is estimated to be <0.02 V nm^−1^, much smaller than the field we are interested in (∼0.2–1.1 V nm^−1^). As a result, the effect of finite source-drain bias on the vertical displacement field is negligible in this work.

In Fig. 2b, the minimal conductance at the charge-neutrality point is plotted as a function of the displacement field **D** in BP. It increases by ∼5 times at maximum bias field, clearly indicating a reduction of the energy gap. We quantify this bandgap reduction in the following. The minimum conductivity *σ*_m_ at the charge neutrality can be calculated using


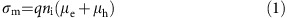


where *q* is the elementary electron charge, *n*_i_ is the intrinsic, thermally excited carrier density for both electrons and holes at charge neutrality, and *μ*_e_ and *μ*_h_ are the mobility for electrons and holes, respectively. If the bandgap reduction is much smaller than the bandgap itself, we can assume that the band structure does not change and hence the carrier mobility remains unchanged. In this case, from the minimum conductivity variation, we can estimate the bandgap reduction, as *n*_i_ is proportional to 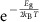 , where *E*_g_ is the bandgap, *k*_B_ is the Boltzmann constant and *T* is temperature (295 K)^19^. The purple dots in Fig. 2c indicate the estimated bandgap shrinkage as a function of the externally applied displacement field, which does not exceed 75 meV and shows a strong nonlinear dependence. Indeed, the reduction is much smaller than the quasi-particle bandgap of ∼4 nm-thick BP (∼7 layers), which should be around 450 meV^9^.

We have performed theoretical calculations using DFT, to explore the bandgap tuning effect in BP thin film. Based on the DFT results, we have further developed a tight-binding model (see details in the Supplementary Note 2, Supplementary Figs 2 and 3, and Supplementary Table 1), in which the interlayer coupling (*δ*) is introduced in the off-diagonal terms of the Hamiltonian and the screen field-induced potential difference (*Δ*) between layers is placed in the diagonal terms. This model can reliably reproduce DFT results for few-layer BP and enable us to efficiently calculate much thicker BP that is beyond the DFT capability. As shown in Fig. 2c, our experimental results measured on a 4 nm-thick BP sample (approximately seven layers nominally) fall in between the theoretical five- and six-layer results. Most importantly, the theoretical results capture the nonlinear character of the bandgap evolution as the gating field increases. In short, the bandgap tuning effect arises from the overall potential difference induced by the external gating field. However, for ultra-thin (few-layer) BP films, the potential drop is less than the interlayer coupling (off-diagonal term *δ*) under low bias. Thus, the bandgap tuning exhibits a very strong nonlinear dependence on the external bias field and the tunable range is rather limited with a moderate displacement field up to ∼1 V nm^−1^. On the contrary, our model predicts that, when the thickness is large enough, the total potential difference across the entire BP thin film dominates, leading to more effective and almost linear dependence of the tuning with the applied bias field as shown in Fig. 2c for thicker BP (16–19 layers). Although in general for thicker material the bandgap tuning effect should be larger, because the Stark coefficient scales with thickness^17^,^18^, here we reveal the distinctively different tuning properties of BP films whose thicknesses fall into two different categories as shown in Fig. 2c.

### Bandgap tuning in a 10 nm-thick BP film

**Figure 3:**
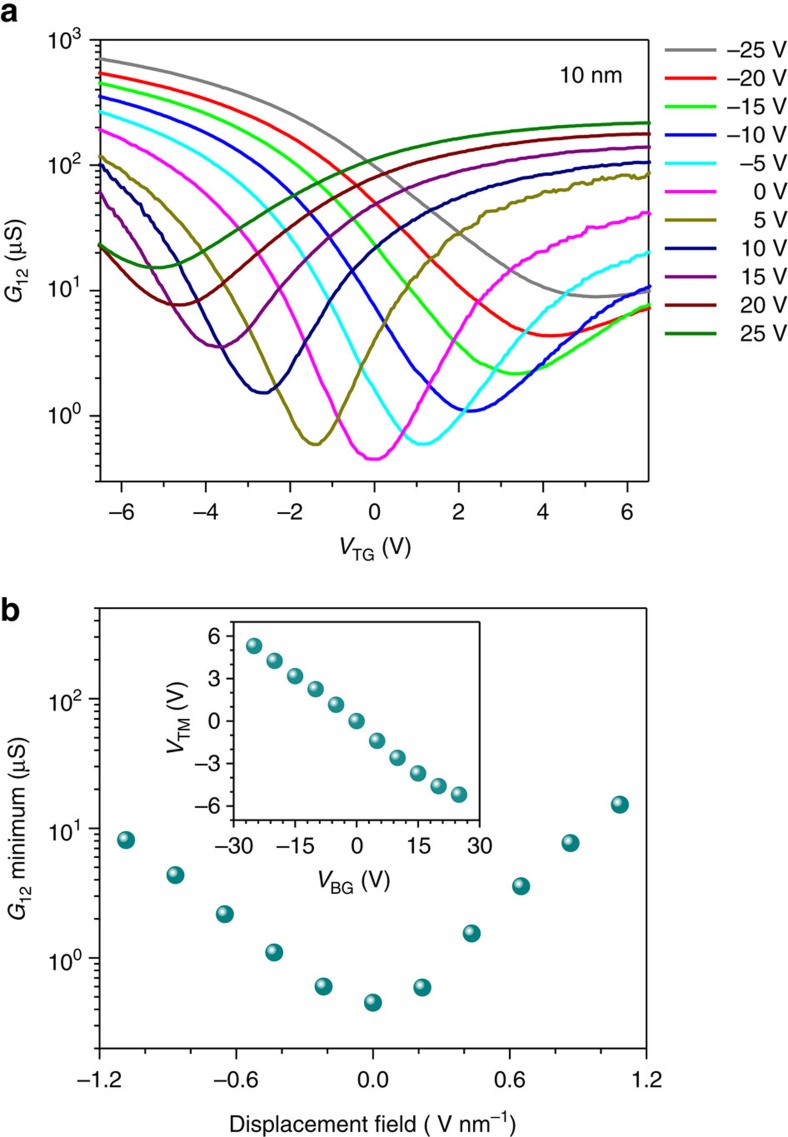
Bandgap tuning in a 10  nm-thick BP film. (**a**) The 10 nm-thick BP film conductance as a function of top gate bias (*V*_TG_) at different static back gate biases (*V*_BG_) from −25 to 25 V. (**b**) The minimum conductance at the charge-neutrality point as a function of external displacement field. Inset: the top gate bias at which the minimum conductance occurs (*V*_TM_) as a function of the back gate bias (*V*_BG_).

**Figure 4:**
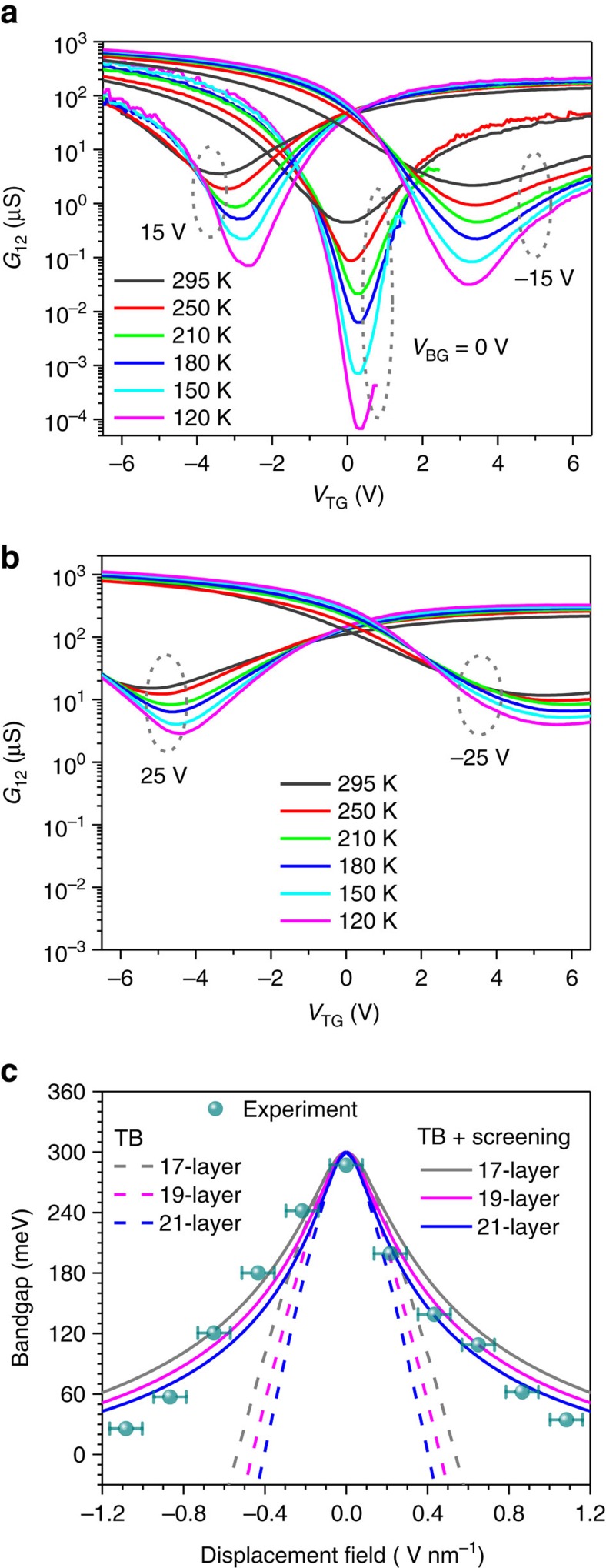
Bandgap determination in 10 nm-thick BP using temperature-dependent measurements. (**a**) The conductance of the 10 nm-thick BP film as a function of top gate bias (*V*_TG_) at different temperatures from 120 to 295 K. The curves at three different back gate biases are plotted (*V*_BG_=0, −15 and 15 V). The bandgap is measured through the temperature-dependent minimum conductivity at the charge-neutrality point. (**b**) Conductance measurement curves similar to those in **a** at two different back gate biases (*V*_BG_=−25 and 25 V). (**c**) Dash (solid) lines: the calculated bandgap tuning properties for BP films consisting of 17, 19 and 21 layers using the tight-binding (TB) model without (with) additional bandgap-dependent dielectric screening effect. Dark cyan dots: the measured bandgap tuning for a 10 nm-thick BP film (∼19 layers). The error bars show the s.d., which accounts for the uncertainty in determination of displacement field due to the minor shift of *V*_TM_ at different temperatures.

Therefore, to extend the bandgap tuning range, we further fabricated the dual-gate transistors with BP channel thickness of ∼10 nm. The conductance measurement results similar to those in 4 nm-thick transistors are plotted in Fig. 3a. The minimum conductance at the charge-neutrality point as a function of the external displacement field is plotted in Fig. 3b. In this transistor, the minimum conductance at the charge neutrality increases by around 40 times as the external bias is maximized, indicating a bandgap reduction significantly >75 meV. In this case, direct extraction of the bandgap variation from the minimum conductance at different biasing field may not be optimal, as the bandstructure itself can vary as the biasing changes the bandgap significantly. In this transistor, we measured the temperature dependence of the minimum conductance to determine the energy gap. Using this method, we not only eliminate the effect of bandstructure change on bandgap determination, but also measure the bandgap directly. The measurement results for the back gate biases of −15, 0 and 15 V are shown in Fig. 4a. In Fig. 4b, similar curves for the back gate biases of −25 and 25 V are shown. The detailed bandgap extraction procedures and the typical fitting curves are presented in the Supplementary Note 3 and Supplementary Figs 4 and 5. Below 120 K, the BP film at zero bias becomes highly insulating and it is not feasible to measure its conductance. The minimum conductance at zero bias varies by around four orders of magnitude from 120 to 295 K as shown in Fig. 4a, from which a bandgap of 290 meV is extracted. This value is close to the established bulk BP bandgap of ∼300 meV, indicating the accuracy of the approach used here. The slight discrepancy can be due to the intrinsic fitting errors of this approach, as the bandgap of BP itself does change by tens of meV as the temperature varies from 300 to 120 K. When the applied displacement field increases from 0 to 1.1 V nm^−1^ (*V*_BG_ positive and *V*_TG_ negative), the bandgap is measured to decrease from 290 to 30 meV continuously, as shown in Fig. 4c. Reversing the direction of the displacement field leads to similar reduction of BP bandgap. When the temperature changes, the top gate bias at which the minimum conductance occurs, *V*_TM_, varies slightly (<0.5 V). We hence introduced an error bar to account for this uncertainty in external biasing displacement field in Fig. 4c.

In Fig. 4c, we also plot the calculated bandgap tuning results using tight-binding model built on DFT results for BP thickness from 9 to 11 nm (∼17 to 21 layers), as shown by the dashed lines. It is clear that DFT predicts a faster bandgap tuning rate as a function of the biasing field. This discrepancy is not surprising, because DFT is known for its deficiency to capture screening effects between electrons and subsequent dielectric function. Unlike the case of 4 nm samples, in which the bandgap variation (∼75 meV) is smaller compared with the bandgap itself (the measured bandgap of 4 nm-thick BP is in fact around 470 meV as shown in Supplementary Fig. 6), the widely changed bandgap in the 10 nm BP makes it necessary to include the variation of dielectric screening beyond DFT. Using the random-phase approximation^20^, in which the dominant contribution to the dielectric constant is from the interband transition around the bandgap, we introduced a gap-dependent dielectric constant 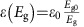 in our tight-binding model, where *ɛ*_0_ is the dielectric constant of pristine BP, *E*_g0_ is the bandgap of pristine BP (∼300 meV in 10 nm-thick film) and *E*_g_ is the bandgap under bias, which can be obtained self-consistently. This model reflects the enhanced dielectric screening when the bandgap is reduced. We have self-consistently calculated bandgaps for BP with thickness from 17 to 21 layers and the results are also plotted in Fig. 4c. The detailed calculation procedures are also presented in Supplementary Note 2. The measured bandgap of 10 nm-thick BP under bias in general agrees well with the self-consistent tight-binding model taking into account the additional dielectric screening. At vertical displacement field above 0.8 V nm^−1^, it seems that the theoretical results underestimate the bandgap reduction, probably due to the oversimplified model for the dielectric constant. Moreover, the measured bandgap tuning results show slight asymmetry as the displacement field switches direction. This is because there are minor uncertainties in the determination of both the bandgap itself and the displacement field. The curve fitting process leads to minor errors in bandgap determination. Moreover, due to the minor device hysteresis in gate bias scans, there is some uncertainty in the determination of displacement field, as indicated by the error bars in Fig. 4c.

## Discussion

In principle, the bandgap tuning effect can be even larger in thicker BP thin films. However, the physical picture above is only valid when the free carrier screening length is longer or comparable to the thickness of BP thin film. In this case, the top and back biases at opposite polarities can effectively compensate the free carriers introduced by each other, leading to an insulating state. In the Supplementary Note 4 and Supplementary Fig. 7, we plot the measured conductance of a 23 nm-thick BP film using the four-probe scheme. It is clear that the doping compensation picture described above does not apply to such thick BP films. As shown in the Supplementary Note 4 and Supplementary Fig. 7, there the top and back gates largely modify the conductance of the top and bottom BP channels, respectively. Therefore, the internal electric field is no longer uniform; the field-induced electrons and holes near the surfaces^21^ will screen the applied electric field. The linear relationship between *V*_TM_ and *V*_BG_ no longer exists and the thick BP film can no longer be regarded as a material system with a universal bandgap. For BP thinner than 4 nm, the bandgap tuning effect is in fact very weak, as shown in Supplementary Note 5 and Supplementary Fig. 8 in which the results on a ∼2.5 nm-thick BP under bias are presented. As a result, a film thickness of 10–15 nm may be optimal for optoelectronic applications. The required field to tune the bandgap of thinner samples can be considerably larger and thinner samples interact with light less strongly. On the contrary, in thicker samples free carrier screening can render the electric field within BP non-uniform, leading to reduced tuning efficiency.

We want to emphasize that this work represents the demonstration of bandgap tuning in thin-film layered materials whose untuned properties are close to the bulk limit. This is particularly important for future device applications, as thin-film materials interact with light more strongly compared with mono- or few-layer materials and the carrier transport in thin film is much less susceptible to environment. Moreover, tuning of bandgap in thin-film BP exhibits distinctively different properties compared with widely explored TMDC bilayers^22^,^23^,^24^. In bilayer TMDCs such as MoS_2_, due to the weak interlayer electronic state coupling, the bandgap can be tuned effectively and linearly with biasing field, as the interlayer potential difference induced by the external field dominates even in bilayer TMDCs^24^. Although vertical electric field changes the bandgap, the optical transitions are hardly affected by the tuning^24^. That is because the wave functions of the conduction band minimum and the valence band maximum are largely separated by the vertical electric field due the weak interlayer coupling in bilayer MoS_2_, leading to the much reduced optical transition matrix elements. The optical measurement primarily picks up the transition between the conduction and valence states localized in the same layer, and this energy is nearly field-independent. On the contrary, BP is a direct bandgap material regardless of its layer number or applied field. Moreover, the wave functions of the conduction band minimum and valence band maximum of thin-film BP are expected to exhibit substantial overlap even under bias due to the strong interlayer electronic state coupling. This character is of particular importance for optical properties, because the overlap gives rise to significant dipole oscillator strength, implying that the optical emission/absorption at the direct bandgap edge can be tuned by the gating-field approach.

In fact, even before the exploration of bilayer TMDs, bandgap opening in bilayer graphene using a vertical electric field has been extensively studied^25^,^26^,^27^,^28^,^29^,^30^,^31^,^32^,^33^. Here the tuning of the bandgap of BP also has distinctively different properties if compared with bilayer graphene. First, it is not feasible to realize bandgap tuning in thin-film graphite due to strong free carrier screening effect, arising from its metallic nature. Moreover, in general experimentally measured transport bandgaps in biased bilayer graphene are much less than the theoretical predictions and the values determined by optical experiments^29^,^30^,^31^,^32^,^33^. This is mainly because the spatial fluctuations of the gating field due to the interfacial charges and charges trapped in gate dielectrics lead to some ungated or weakly gated areas^33^. Although they probably represent only a small fraction of the bilayer sample area, these ungated or weakly gated areas are close to metallic and have large conductance. As a result, carrier transport is significantly affected by these highly conductive ‘leakage' paths^33^, leading to discrepancy in theory and experiments. In BP thin film, although it is highly likely that there are also such ungated or weakly gated areas, those areas are semiconducting and the transport properties under the bias are dominated by the efficiently gated areas in which the bandgap is significantly reduced. As a result, our transport measurement results agree with theoretical values well.

Finally, we want to comment on the possible role of interfacial state density (*D*_it_) on our bandgap measurement results. As discussed in Supplementary Note 6 and Supplementary Fig. 9, the interface states can affect the subthreshold slope significantly. However, as long as those states do not participate in current transport themselves, they do not affect the minimum conductivity. As a result, the *D*_it_ has negligible effect on the bandgap determination in this work.

In summary, we demonstrate the unique bandgap tuning properties of thin-film BP and further show that the bandgap of a 10 nm-thick BP can be continuously tuned from ∼300 meV to below 50 meV, with a moderate field readily achieved using regular dielectrics. This demonstration may significantly extend the operational wavelength range of BP optoelectronic devices, making it a technologically relevant material for tunable infrared applications beyond the cutoff wavelength of pristine BP (∼3.8 μm). The concept of bandgap tuning may also be leveraged to construct novel electronic devices. Interestingly, this demonstration may further enable the exploration of electrically tunable, BP-based topological insulators and topological nodal semimetals^17^,^18^,^34^.

## Methods

### Fabrication of dual-gate BP transistors

The fabrication of the devices started with the exfoliation of BP thin films from bulk crystals onto 90 nm SiO_2_ on a silicon substrate in an argon-filled glovebox with both oxygen and water concentrations well below one part per million (1 p.p.m.). The thickness of BP flake was determined by AFM and high-resolution transmission electron microscopy measurements. The poly (methyl methacrylate) resist layer was spun on wafer and then patterned for metallization using a Vistec 100 kV electron-beam lithography system. Chromium/Gold (3/30 nm) were then evaporated and the following lift-off process in acetone formed the source, drain and voltage probe contacts on BP flakes. The 24 nm-thick Al_2_O_3_ top-gate dielectric was formed by atomic layer deposition at 150 °C. Titanium/Platinum (1/10 nm) top-gate electrodes were fabricated using the same metallization procedure as discussed above. The top-gate electrode was designed to cover the entire transistor to ensure the full control of the channel.

### Conductance measurements using four-probe scheme

All the electrical measurements were performed using an Agilent B1500A semiconductor parameter analyser together with a Keithley 2612B source meter in a Lakeshore cryogenic probe station with six probe arms. The static back-gate biases were applied using the source meter, whereas the conductance as a function of top gate bias was measured using the Agilent parameter analyser. The drain bias was set to be as low as 100 mV, to minimize the drain-induced doping non-uniformity across the channel, thus ensuring the accurate determination of the conductance minima. The conductance of BP between probes 1 and 2 was obtained through dividing the channel current by the voltage drop between them. The impact of the contact resistance is eliminated by this four-probe scheme. These Al_2_O_3_-covered BP transistors showed excellent stability. In fact, the measurements usually involved hundreds of scans and multiple temperature cycles. Most of the devices remained operational without noticeable performance degradation after the measurements.

### Data availability

The data that support the findings of this study are available from the corresponding authors upon request.

## Additional information

**How to cite this article:** Deng, B. *et al*. Efficient electrical control of thin-film black phosphorus bandgap. *Nat. Commun.* **8,** 14474 doi: 10.1038/ncomms14474 (2017).

**Publisher's note**: Springer Nature remains neutral with regard to jurisdictional claims in published maps and institutional affiliations.

## Supplementary Material

Supplementary Information Supplementary Figures, Supplementary Notes, Supplementary Table and Supplementary References
