# Supplementary material for: Efficient electrical control of thin-film black phosphorus bandgap
Source: Nat Commun. 2017 Apr 19;8:14474. doi: 10.1038/ncomms14474 (PMC5399305; doi:10.1038/ncomms14474)
Supplement: Supplementary Information — Supplementary Figures, Supplementary Notes, Supplementary Table and Supplementary References [file ncomms14474-s1.pdf]

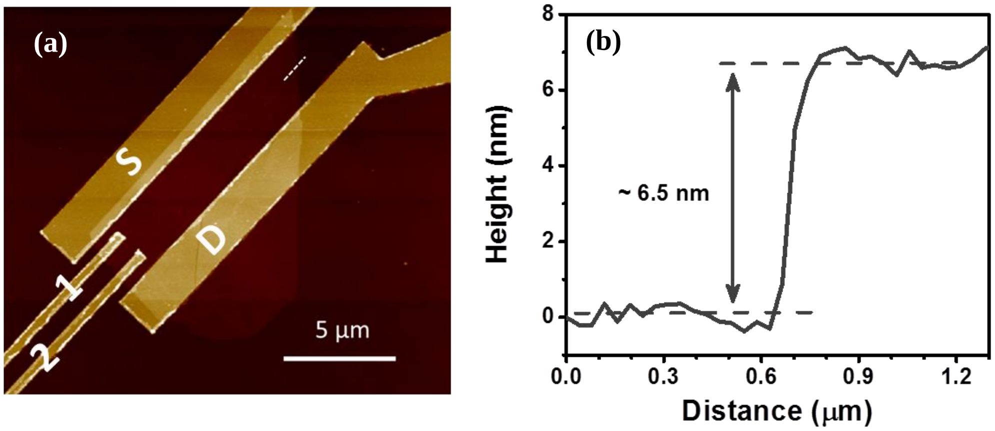

Supplementary Figure 1. (a) An AFM image of the device after the formation of the contact electrodes and the top gate dielectric Al_2O_3. **(b)** A line scan performed along the white dashed line in **(a)**.

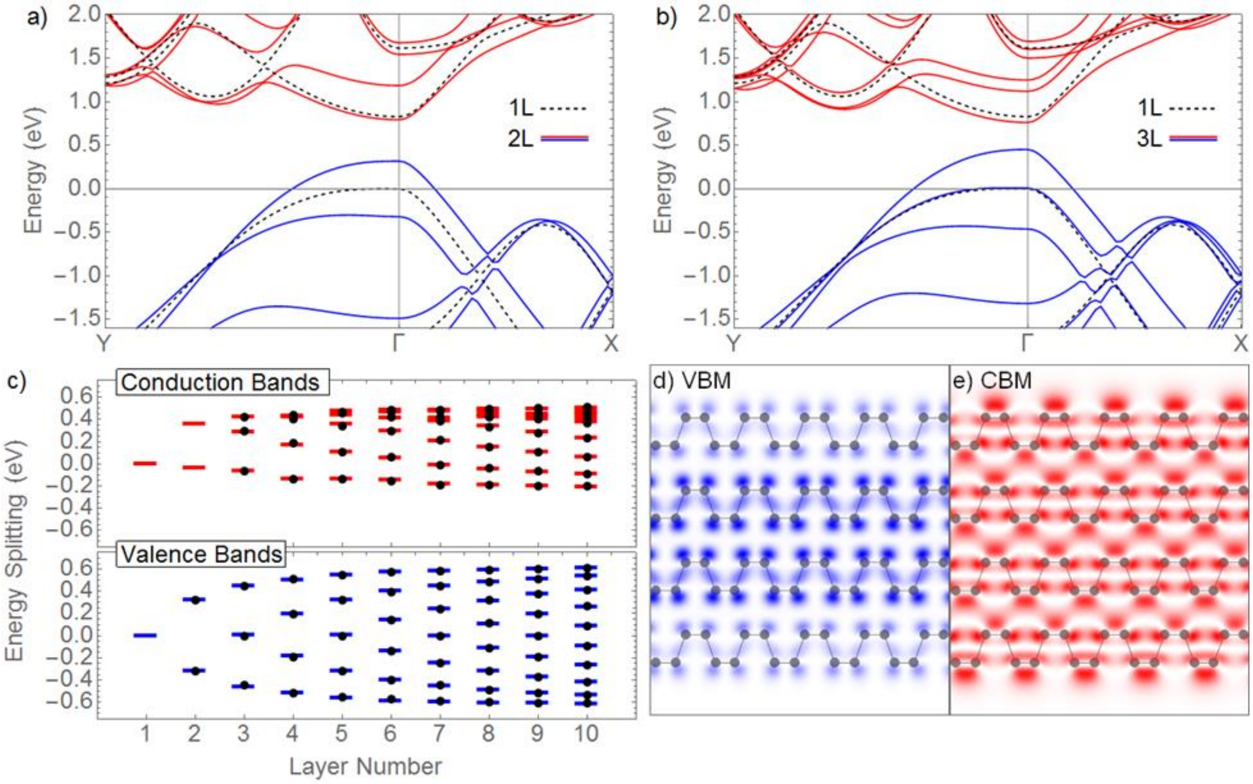

Supplementary Figure 2. (a) The DFT-calculated band structure of 2-layer BP compared with monolayer BP. **(b)** The DFT-calculated band structure of 3-layer BP compared with monolayer BP. **(c)** The evolution of the energy of the valence band maximum (VBM) and conduction band minimum (CBM) states as the number of layers is increased. Colored lines represent direct DFT results and points are the fitted results using TB model. **(d)** and **(e)** The wave functions of the VBM and CBM states for 4-layer BP, respectively.

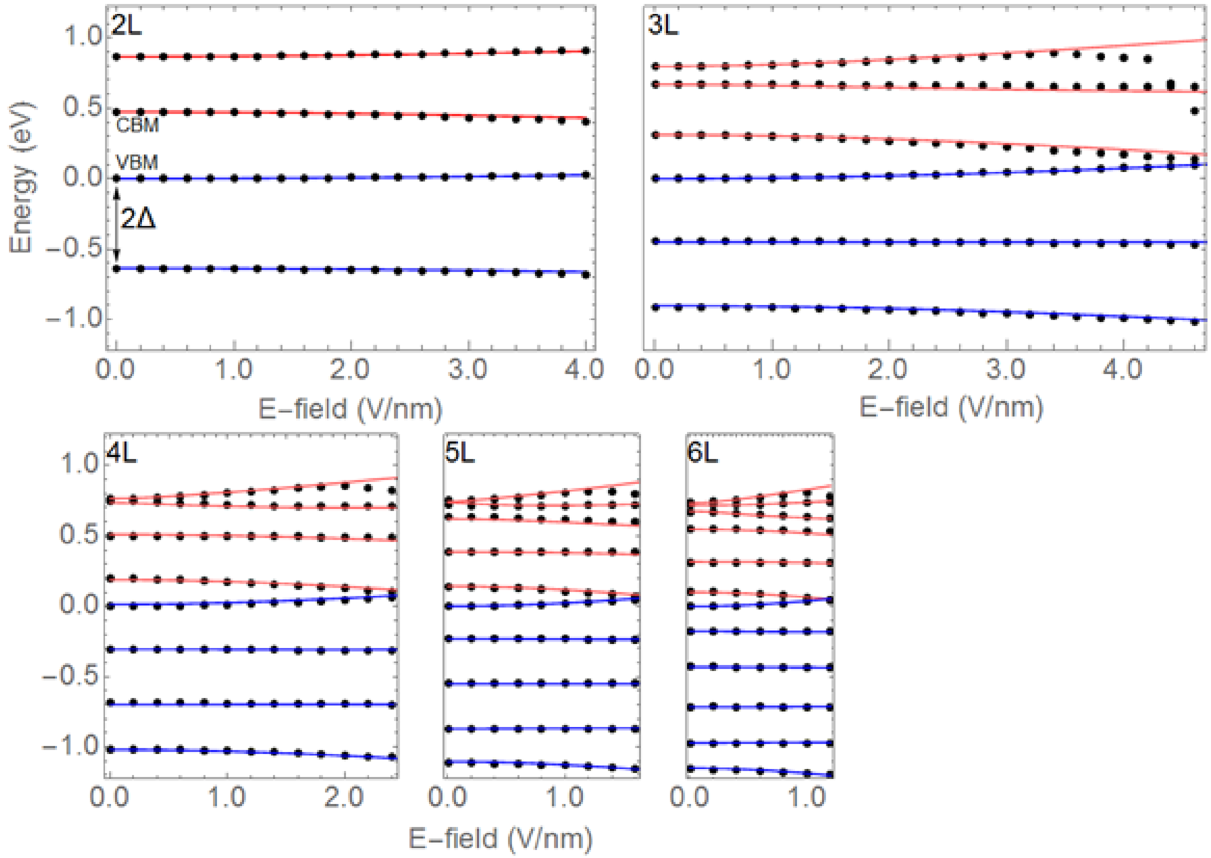

Supplementary Figure 3. The DFT-calculated energies of the VBM and CBM states for 2 to 6-layer BP as a function of applied electric field. The points are from DFT calculations, and the lines are from diagonalizing the model Hamiltonian. DFT and tight binding results agree well. Again, we want to emphasize that the values of these DFT calculated bandgaps are substantially underestimated.

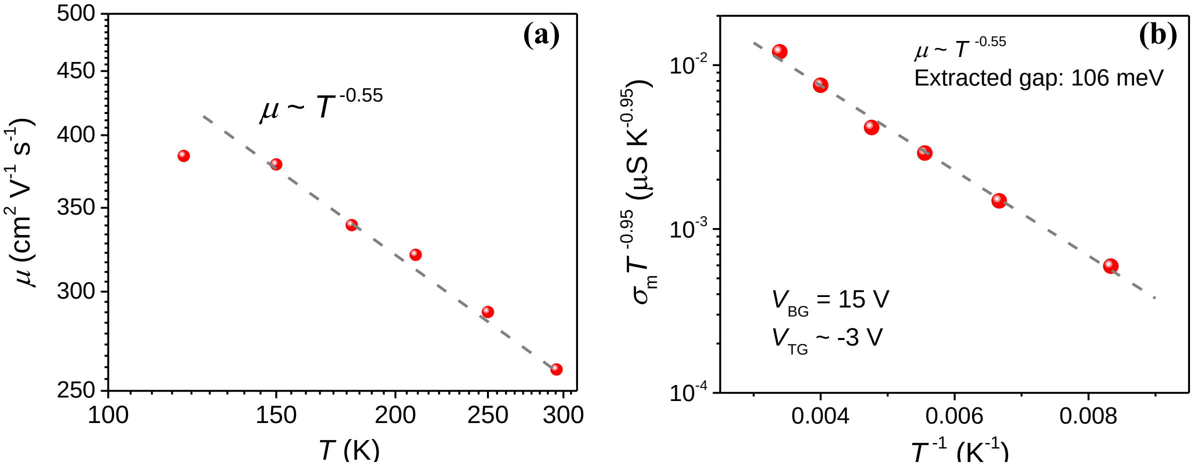

Supplementary Figure 4. (a) The hole mobility as a function of temperature. At 120 K, the mobility deviates from the fitting slightly because phonon scattering is no longer the mobility limiting factor. **(b)** A typical fitting curve to determine the bandgap of the BP thin film under the bias (V_{BG} = 15 V and V_{TM} ≈ -3 V) using $\mu \propto T^{-0.55}$. Red dots: measured results. Dash line: linear fitting curve.

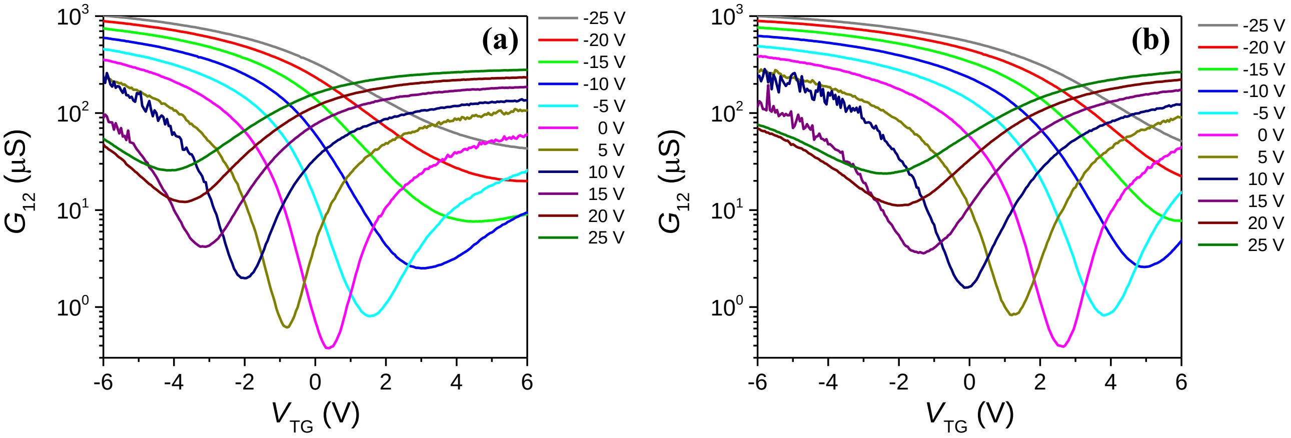

Supplementary Figure 5. (a) A 10-nm-thick BP film conductance as a function of top gate bias (V_{TG}) at different static back gate biases (V_{BG}) from -25 to 25 V. The top gate is scanned from negative to positive voltage. **(b)** The same group of conductance curves as in (a) when the top gate is scanned from positive to negative voltage.

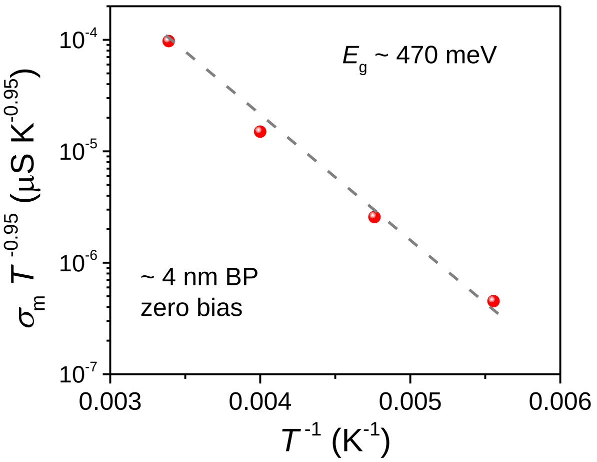

Supplementary Figure 6. A typical fitting curve to determine the bandgap of 4-nm-thick BP under zero bias.

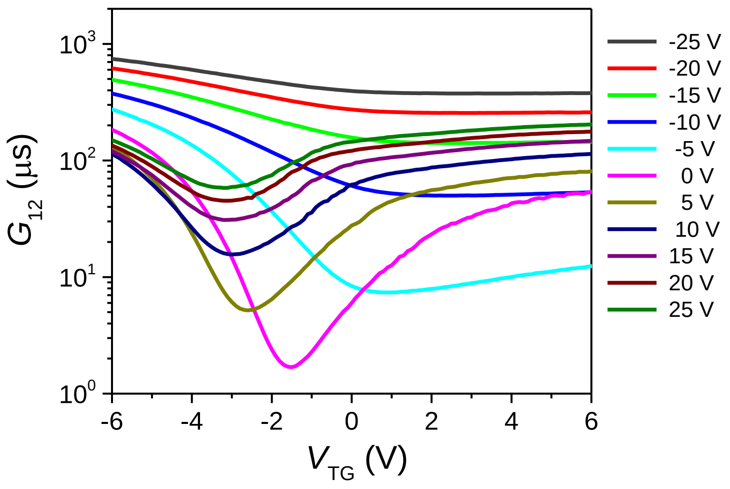

Supplementary Figure 7. The overall conductance G_{12} of a 23-nm-thick BP thin film as a function of top gate bias for fixed back gate bias from -25 to 25 V.

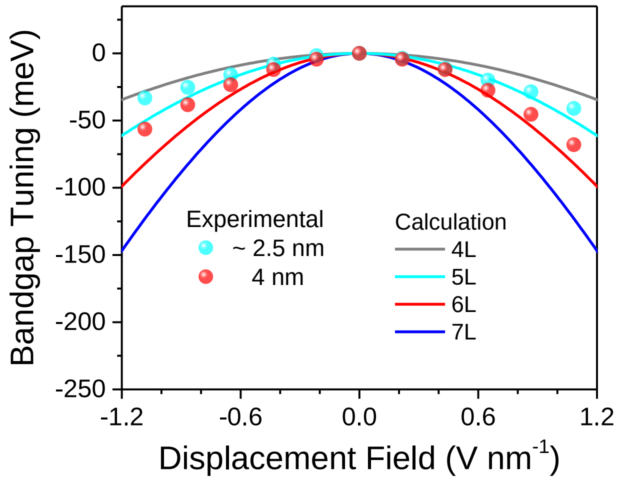

Supplementary Figure 8. The bandgap tuning results of a ~2.5-nm-thick BP thin film as a function of the displacement field. For comparison, experimental results from 4-nm-thick film and theoretical results (4 to 7 layers) are also plotted.

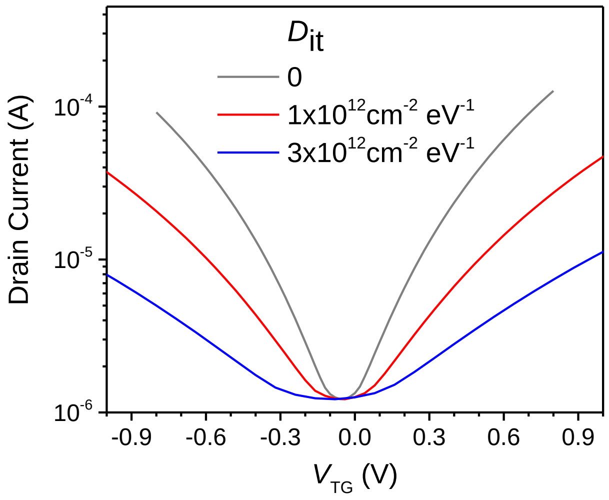

Supplementary Figure 9. Plots of simulated drain current versus gate voltage for a BP transistor similar to those utilized in experiments in this work. The drain-to-source voltage is -0.1 V. The current is calculated for interface state density, D_{it}, values 0, 1×10^{12} and 3×10^{12} cm^{-2} eV^{-1}.

Layers	2	3	4	5	6	7	8	9	10	Bulk
δ_1^{cond}	196	170	176	172	163	165	163	161	159	141
δ_2^{cond}		-76	-82	-73	-89	-93	-94	-95	-97	-85

Supplementary Table 1. The parameters for the conduction band model Hamiltonian obtained by fitting to DFT calculations. All values are in meV.

Supplementary Note 1: Thickness determination of black phosphorus thin film

We determine the thickness of the black phosphorus (BP) thin film using the standard atomic force microscope (AFM). Supplementary Figure 1a shows a typical AFM image of device after the formation of the contact electrodes (source, drain contacts and voltage probes 1 and 2) and the atomic layer deposition of an Al_2O_3 gate dielectric. The measured thickness of this BP flake is about 6.5 nm, as shown by the line scan in Supplementary Figure 1b. Here the Al_2O_3 deposition does not affect the measurement accuracy since it is deposited uniformly on both the substrate and the BP film.

Similar to other elemental semiconductors such as silicon (Si) and germanium (Ge), it is well known that BP can oxidize. In order to accurately determine the BP thickness, separate from the possible surface oxide layer, we further characterized the sample using cross-section high-resolution transmission electron microscopy (HRTEM) and energy dispersive X-ray spectroscopy (EDX) to determine the intrinsic BP thickness. The results are summarized in Figs. 1c and 1d in the main text. By comparing the AFM and HRTEM results, here we show that the thickness determined with AFM includes 2 to 3 nm phosphorus oxide thickness.

Supplementary Note 2: Modeling of the BP bandgap under bias based on tight binding (TB) model

The bandgap reduction in multi-layer BP can be described by considering a tight-binding model which contains the energies of monolayer BP and coupling terms which describe the interlayer electronic state coupling. This model is motivated by examining the set of bands around the band gap for few-layer BP. Supplementary Figures 2a and 2b show the band structure for 2-layer and 3-layer BP computed based on the density functional theory (DFT), respectively. It is clear that adding layers produces additional bands which look like the monolayer bands but are split off from each other due to interlayer coupling, like a particle tunneling between multiple wells. We want to emphasize that the values of these DFT calculated bandgaps are substantially underestimated. Fortunately, we are interested in the variations of bandgaps according to the applied gating field. Meanwhile, in final comparison with measurements in the main text, we include the many-electron self-energy corrections for correcting absolute bandgap values.

First, we discuss the model without an external electric field. The model Hamiltonian for n-layer BP can be written in matrix form as:

$$H_{\mathrm{n}} = \begin{pmatrix} E_0 & \delta_1 & \delta_2 & \cdots \\ \delta_1 & E_0 & \delta_1 & \cdots \\ \delta_2 & \delta_1 & E_0 & \cdots \\ \vdots & \vdots & \vdots & \ddots \end{pmatrix}_{\mathrm{n}\times\mathrm{n}} \tag{1}$$

where the diagonal term E_0 is the energy of monolayer BP and the off-diagonal terms δ_{i} describe the coupling between layers. When the subscript i=1, δ_1 is the nearest neighboring interlayer coupling; when the subscript i=2, δ_2 is the second nearest neighboring interlayer coupling. In principle, the Hamiltonian is k dependent, however since we are only interested in the valence

band maximum (VBM) and conduction band minimum (CBM) states, we set $k = 0$, simplifying the model significantly.

The values of the parameters were obtained by fitting the model to DFT calculations. For n-layer BP, the original VBM/CBM is split into n bands, which are fit to the n-1 parameters (the off-diagonal terms δ_{i}) in the model. This direct fitting approach was performed for up to ten layers, and the comparison of the fitted model to DFT is shown in Supplementary Figure 2c. For the valence bands, the value of the nearest-neighbor term δ_1, is 318 meV. The terms beyond δ_1 were almost zero, and are treated as zero in the model. For the conduction bands, the coupling terms depend on the number of layers, but converge to a bulk limit, which is extrapolated and used for $n > 10$. For 10-layer to 20-layer BP, we assume that their local interlayer coupling is nearly the same as the bulk values. Thus we set $\delta_1^{\mathrm{cond}} = 141$ meV and $\delta_2^{\mathrm{cond}} = -85$ meV. For the conduction band the terms beyond δ_2 were found to be small enough to ignore. The parameters for the conduction band are summarized in Supplementary Table 1.

To explain the layer dependence and additional coupling terms in the conduction bands we compare the VBM and CBM wave functions for 4-layer BP in Supplementary Figures 2d and 2e. It is clear that the VBM state is much more localized than the CBM state. The CBM state has considerable amplitude in the interlayer region, as well as extending from the surfaces. This leads to the second-neighbor coupling. Another feature of second-neighbor coupling is the asymmetric splitting of the CBM energies. From Supplementary Figure 2c we see that the valence bands are split symmetrically around the monolayer energy, while conduction band energy split is highly asymmetric. The single off-diagonal term in the valence band model controls the strength of the split, and additional terms in the conduction band model lead to asymmetric split.

Our model then contains effectively three parameters: δ_1^{val}, δ_1^{cond} and δ_2^{cond}, which are obtained by fitting to DFT calculations for up to 10 layers. This model describes the band gap evolution with respect to the layer number. To obtain the band gap in the presence of an out of plane electric field, an additional term is added, which shifts the potential of each layer due to the electric field. The models of conduction bands and valance bands can be written as:

$$H_{\mathrm{n}}^{\mathrm{c}} = \begin{pmatrix} E_0^{\mathrm{c}} & \delta_1^{\mathrm{cond}} & \delta_2^{\mathrm{cond}} & \cdots \\ \delta_1^{\mathrm{cond}} & E_0^{\mathrm{c}} + \Delta & \delta_1^{\mathrm{cond}} & \cdots \\ \delta_2^{\mathrm{cond}} & \delta_1^{\mathrm{cond}} & E_0^{\mathrm{c}} + 2\Delta & \cdots \\ \vdots & \vdots & \vdots & \ddots \end{pmatrix}_{\mathrm{n\times n}} \quad \text{and } H_{\mathrm{n}}^{\mathrm{v}} = \begin{pmatrix} E_0^{\mathrm{v}} & \delta_1^{\mathrm{val}} & 0 & \cdots \\ \delta_1^{\mathrm{val}} & E_0^{\mathrm{v}} + \Delta & \delta_1^{\mathrm{val}} & \cdots \\ 0 & \delta_1^{\mathrm{val}} & E_0^{\mathrm{v}} + 2\Delta & \cdots \\ \vdots & \vdots & \vdots & \ddots \end{pmatrix}_{\mathrm{n\times n}} \quad (2)$$

where $\Delta = \frac{E^{\mathrm{ext}}}{\epsilon} * d$, E^{ext} is the external electric field, $\epsilon = 8.3$ is the dielectric constant of intrinsic BP [1], and $d = 0.53$ nm is the layer-layer distance. Here we emphasize that in our model E^{ext} is the external field in vacuum. In our devices, the dielectric constant of the gate stack has to be taken into account. The potential on each layer is then shifted by an amount Δ, which depends on the layer-layer distance and screened electric field in BP. Note that the addition of an electric field to the model requires no additional tunable parameters. Supplementary Figure 3 shows the results obtained by tight binding model and DFT for 2-6 layers under bias; the tight binding model agrees with DFT extremely well. In the Fig. 2c of the main text, the calculated bandgap results of 4, 5, 6, 7, 16, 17, 18, and 19 layers of BP under bias using the tight binding model are plotted.

Although the tight binding model accurately reproduces the results of DFT calculations, it assumes the dielectric constant is the same for all layer numbers under all electric field conditions. Realistically, as the band gap decreases, the system should become more metallic, and the dielectric constant should increase. This effect is not significant for BP with a thickness of ~4 nm since the bandgap tuning is less than 100 meV and the original bandgap is estimated to be around 450 meV. However, for ~10-nm BP, this effect can be significant since the tuning exceeds 250 meV and the original bandgap is ~300 meV. To account for this, the dielectric constant in the expression for Δ is taken to be $\varepsilon\left(E_{\mathrm{g}}\right)=\varepsilon_0 \frac{E_{\mathrm{g0}}}{E_{\mathrm{g}}}$ in our tight-binding model, where ε_0 is the dielectric constant of pristine BP [2], E_{g0} is the bandgap of pristine BP (~290 meV in 10-nm-thick film), and E_{g} is the bandgap under bias, which can be obtained self-consistently. The additional screening reduces the band gap tuning, as can be seen from Fig. 4c in the main text.

Supplementary Note 3: Determination of the BP bandgap under bias

At each back-gate bias, we performed temperature-dependent measurements to determine the minimum conductance as a function of temperature. When the top gate bias reaches V_{TM}, the BP sample is at its overall intrinsic state and therefore we have:

$$\sigma_{\mathrm{m}} = q n_{\mathrm{i}}(\mu_{\mathrm{e}} + \mu_{\mathrm{h}}) \qquad (3)$$

where σ_{m} is the minimum conductivity, q is the elementary charge, n_{i} is the intrinsic carrier density for both electrons and holes, and μ_{e} and μ_{h} are the mobility for electrons and holes, respectively. The minimum conductivity is a function of temperature primarily because we have [3]:

$$n_{\mathrm{i}} \propto \left(\frac{m_{\mathrm{de}} m_{\mathrm{dh}}}{m_0^2}\right)^{3/4} T^{3/2} \mathrm{e}^{-E_{\mathrm{g}}/2k_{\mathrm{B}}T} \qquad (4)$$

where T is the temperature, m_{de} and m_{dh} are density of state (DOS) masses for electron and holes, respectively, m_0 is the free electron mass, E_{g} is the material bandgap, and k_{B} is the Boltzmann constant. Moreover, both electron and hole mobility depend on the temperature. In BP thin films, previous experiments show that the carrier mobility has a temperature dependence of T^{γ} [4-6], where γ varies from 0.5 to 1, depending on the sample fabrication processes, the kind of mobility (Hall or field-effect), carrier concentration and local environment. In our ~10 nm thick BP transistors, the field effect mobility is measured to have a γ between 0.5 to 0.6. A typical mobility as a function of temperature curve for holes is shown in Supplementary Figure 4a. Using γ = 0.55, we have $\frac{\sigma_{\mathrm{m}}}{T^{0.95}} \propto e^{-E_{\mathrm{g}}/2k_{\mathrm{B}}T}$, based on Supplementary Equation 3 and Supplementary Equation 4 above. Therefore, the bandgap can be determined from the slope of the $\ln(\frac{\sigma_{\mathrm{m}}}{T^{0.95}})$ v.s. $(\frac{1}{T})$ curve. A typical fitting curve at V_{BG} of 15 V (V_{TG} ~ -3 V) is plotted in Supplementary Figure 4b. At the high electric field, the bandgap can be very small. In this case, we replace the Supplementary Equation 4 above by the original Fermi-Dirac integral and performed the fitting again. The difference in the bandgap values at all biases is within 5%. In addition, we also determined the bandgap assuming γ =1. In this case, we extracted the bandgap from the slope of the $\ln(\frac{\sigma_{\mathrm{m}}}{T^{0.5}})$ v.s. $(\frac{1}{T})$ curve. The difference in the extracted bandgap values is

always within 14 meV using γ of 0.5 and 1 at all the biasing fields in this work. Here we want to emphasize that the mobility of intrinsic carriers (electrons and holes) in Supplementary Equation (3) can be different from the field effect mobility measured from the transistor transfer characteristics, since the intrinsic carrier density is much lower. However, here we show that the intrinsic carrier density n_i itself plays a decisive role in the determination of the bandgap, due to its exponential dependence on the temperature. The intrinsic mobility and the temperature is expected to have power-law dependence, which has insignificant effect on the extracted bandgap.

In all the measurements results reported in the main text, the top gate scanning was performed from negative to positive voltage. There is hysteresis effect in our devices and reversing the scanning direction usually leads the systematic shift of the group of the curves by around 2 V, as shown in Supplementary Figure 5. However, by subtraction of a constant voltage from V_{TM}, the analysis and the conclusion will not be affected, since both the minimum conductance and the displacement do not change appreciably when the scanning direction changes.

Here we also plot the fitting curve to determine the bandgap of 4-nm-thick BP using temperature-dependent measurements in Supplementary Figure 6. As discussed in in Supplementary Note 1, this BP thickness measured using AFM is ~6.5 nm. Indeed the extracted bandgap (~470 meV) is close to the theoretical prediction of ~450 meV as discussed in the main text.

Supplementary Note 4: A 23-nm-thick BP film under bias

We also performed similar conductance measurements on thicker BP thin films. Supplementary Figure 7 denotes the conductance of a 23-nm-thick BP film under bias using the 4-probe scheme. Here the results are distinctively different from those in Figs. 2a and 3a in the main text. First, when the back gate bias is negative (-25 to -10 V), no obvious conductance minimum is observed. Second, when the back gate bias is positive (5 to 25 V), the V_{TM}, the top gate bias at which the conductance minimum occurs, is almost independent of the back gate bias. Both phenomena indicate that in thick BP thin-film, the electrostatic doping introduced by top and bottom gate biases cannot be compensated even if they are of the opposite polarity, because the free carriers screen the displacement field introduced by the gates. In this case, the BP channel can be approximately regarded as two rather independent channels controlled by the back and top gates, respectively. When the back gate bias is negative, holes accumulate in the bottom BP channel. Due to the high hole mobility, the bottom channel has a large conductance. The top gate mainly modulates the conductance of the top BP channel. Due to the relatively low electron mobility, the top BP channel conductance is low when V_{TG} is positive, leading to the rather small change in overall conductance G_{12} when V_{TG} varies from 0 to 6 V. The overall G_{12} conductance minimum can be observed when the bottom gate bias changes from 0 to 25 V. This is because at zero back gate bias the bottom BP channel conductance is minimized and at positive back gate bias the bottom BP channel conductance is relatively small due to the low electron mobility. Again, due to the free carrier screening effect, the negative top gate bias does not effectively

compensate the electron doping in bottom BP channel, which leads to rather stable V_{TM} when the back gate bias changes from 5 to 25 V.

Supplementary Note 5: A ~2.5-nm-thick BP film under bias

In order to further explore the bandgap tuning effect, a device with a channel thickness of ~2.5 nm (4 to 5 layers) was fabricated and the bandgap tuning results are presented in Supplementary Figure 8. Here the top 2 to 3-nm-thick BP oxide thickness has been subtracted from the AFM thickness measurement result. Indeed, under the biasing displacement field of 1.1 V nm^{-1}, the bandgap tuning is only 40 meV, much smaller than that observed in the 4-nm-thick BP transistor (75 meV). Moreover, the field dependence of bandgap tuning agrees very well with the theoretical calculations. As discussed in the main text and in the Supplementary Note 2, the reduced bandgap tuning is due to the significant reduction in the overall potential difference across the BP film. This observation further confirms the unique thickness dependent bandgap tuning properties of BP.

Supplementary Note 6: The impact of interfacial state density (D_{it})

In this section we describe the expected effect of interface traps on the minimum conductivity. We have performed additional simulations based upon the theoretical approach described in Ref. [7], which has been shown to provide excellent agreement with experimental data on black phosphorus transistors in the subthreshold region. In these simulations, the surface potential, ψ_s, in the center of the channel between source and drain is assumed to change in proportion to the top gate-to-source voltage, V_{TG}, with a constant of proportionality of m, where $m = 1+C_{it}/C_{ox}$. Here we assume the depletion capacitance is zero to simplify the analysis, since the minimum current does not depend upon how fast the gate bias moves the bands. In other words, we simply assume $dV_{TG}/d\psi_s = m$. Here, C_{it} is the interface state capacitance and C_{ox} is the oxide capacitance. C_{it} can be further related to the interface state density, D_{it}, by $C_{it} = q^2 D_{it}$.

The simulation results shown in Supplementary Figure 9 indicate that while the subthreshold slope changes with increasing D_{it}, the minimum current does not change, even when D_{it} is very large (3×10^{12} cm^{-2} eV^{-1}). This result is due to the fact that, to first order, the interface traps only affect the value of m, leading to a “stretch out” of the I-V curve, but otherwise leaving it unchanged.

It should be noted that the interface traps can indirectly influence the channel conductivity through Shockley-Read-Hall (SRH) generation. However, even in this case, the SRH generation current would be proportional to the intrinsic carrier density n_i, and therefore has the same activation energy as the Ohmic conductivity. For these reasons, it is sensible to conclude that D_{it} effects do not impact the field-modulated band gap values determined in the main manuscript.

Supplementary References

[1] Nagahama, T., Kobayashi, M., Akahama, Y., Endo, S. & Narita, S.-i. Optical determination of dielectric constant in black phosphorus. *J. Phys. Soc. Jpn.* **54**, 2096-2099 (1985).

[2] Rudenko, A. N., Yuan, S. & Katsnelson, M. I. Toward a realistic description of multilayer black phosphorus: From GW approximation to large-scale tight-binding simulations. *Phys. Rev. B* **92**, 085419 (2015).

[3] Sze, S. M. & Ng, K. K. Physics of semiconductor devices. Chapter 1. John Wiley & Sons. New Jersey (2007).

[4] Li, L. *et al.* Quantum Hall Effect in black phosphorus two-dimensional electron gas. *Nat. Nanotechnol.* **11**, 593-597 (2016).

[5] Chen, X. *et al.* High-quality sandwiched black phosphorus heterostructure and its quantum oscillations. *Nature Commun.* **6**, 7315 (2015).

[6] Tayari, V. *et al.* Two-dimensional magnetotransport in a black phosphorus naked quantum well. *Nature Commun.* **6**, 7702 (2015).

[7] Haratipour, N., Namgung, S., Oh, S.-H. & Koester, S. J. Fundamental limits on the subthreshold slope in Schottky source/drain black phosphorus field-effect transistors. *ACS Nano* **10**, 3791-3800 (2016).
